# Supplementary material for: Imaging Glycosylation In Vivo by Metabolic Labeling and Magnetic Resonance Imaging
Source: Angew Chem Int Ed Engl. 2015 Dec 3;55(4):1286–90. doi: 10.1002/anie.201509858 (PMC4737346; doi:10.1002/anie.201509858)
Supplement: Supplementary file 1 — Supplementary [file ANIE-55-1286-s001.pdf]

Angewandte Chemie
Eine Zeitschrift der Gesellschaft Deutscher Chemiker

Supporting Information

Imaging Glycosylation In Vivo by Metabolic Labeling and Magnetic Resonance Imaging

André A. Neves, Yéléna A. Wainman, Alan Wright, Mikko I. Kettunen, Tiago B. Rodrigues, Sarah McGuire, De-En Hu, Flaviu Bulat, Simonetta Geninatti Crich, Henning Stöckmann, Finian J. Leeper, and Kevin M. Brindle*

anie_201509858_sm_miscellaneous_information.pdf
anie_201509858_sm_Video_1.avi

Supplementary Information

I General Procedures
II Synthesis
III *In vitro* methods
IV *In vivo* methods
V Ex vivo methods
VI Supplementary video 1
VII References

I - General Procedures

Materials. All reagents were obtained from commercial suppliers (Sigma-Aldrich, Acros, Expedeon, Invitrogen, Fisher Biosciences, Merck, Alfa Aesar) and used without further purification, unless stated otherwise.

Methods. NMR spectra were recorded on Bruker DRX500, Bruker Avance BB, Bruker Avance TCI, Bruker AM400 and Bruker DRX400 instruments. All chemical shifts are quoted in ppm, relative to tetramethylsilane, using the residual solvent peak as a reference standard. All coupling constants are quoted in Hz. Infrared spectra were recorded on a Perkin Elmer Spectrum One (FT-IR) spectrophotometer. High-resolution mass spectra (HRMS) were recorded on a Waters LCT Premier TOF mass spectrometer with electrospray and modular Lockspray interface. Analytical thin layer chromatography (TLC) was carried out on Merck Kieselgel 60 F254 plates with visualization by ultra violet light (254 nm), potassium permanganate and ninhydrin dip. Flash column chromatography was carried out using Merck Kieselgel 60 (230-400 mesh). HPLC was carried out on a Varian ProStar system with UV detection at 254 nm, Supelco semi-prep C18 column, particle size 5 μm. For preparatory HPLC work with TFA, a Zorbax semi-prep C18 column, particle size 5 μm was used. LC-MS analysis was performed on a Waters 2795 system with UV detection at 254 nm, Supelco ABZ+plus column, 3.3cm x 4.6mm, particle size 3 μm. All solvent mixtures are reported as % vol/vol unless stated otherwise. Reagents and solvents were purified using standard means. All other chemicals were used as received unless noted otherwise. Extractive procedures were performed using distilled solvents and evaporation of solvents was performed under reduced pressure. All aqueous solutions used were saturated. Unless stated otherwise, all non-aqueous reactions were carried out under an argon atmosphere using anhydrous conditions and oven-dried glassware. Standard techniques were employed for handling air-sensitive materials. Intermediates p-SCN-Bn-NOTA and DOTA-NHS-ester were purchased from Macrocyclic Inc (Dallas, TX).

II - Synthesis

The synthesis of **1** ($Ac_4GalNAz$) has been described elsewhere.[1] TMDIBO-Lys was synthesized following a procedure that we reported previously.[2]

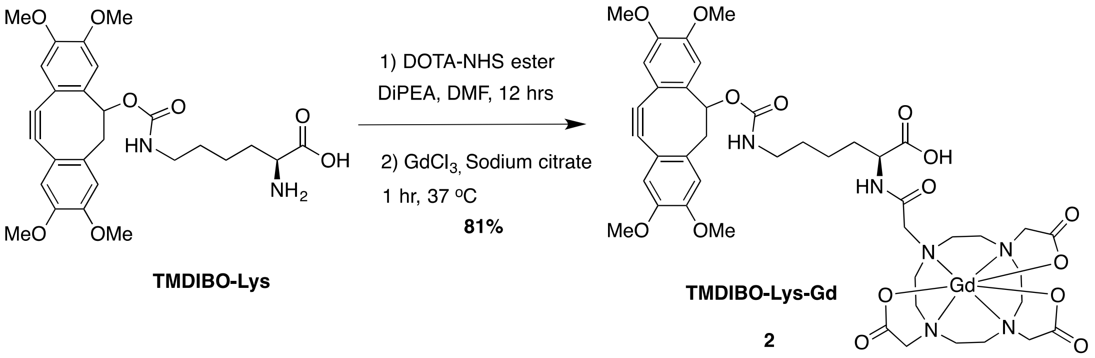

Synthesis of TMDIBO-Lys-Gd First, TMDIBO-Lys was reacted with DOTA-NHS-ester followed by chelation with $GdCl_3$ in a one pot procedure rendering the water soluble 2,2',2''-(10-(2-(((1*S*)-1-carboxy-5-(((((*Z*)-2,3,8,9-tetramethoxy-11,12-didehydro-5,6-dihydrodibenzo[*a*,*e*][8]annulen-5yl)oxy)carbonyl)amino)pentyl)amino)-2-oxoethyl)-1,4,7,10-tetraazacyclododecane-1,4,7-triyl)triacetic acid gadolinium (TMDIBO-Lys-Gd; 81% yield after HPLC purification)

TMDIBO-Lys (100 mg, 0.20 mmol) and DOTA-NHS ester (200 mg, 0.26 mmol) were dissolved in anhydrous DMF (2.7 mL). DiPEA was then added (322 µL, 1.95 mmol) and the reaction mixture was left to stir overnight (LC-MS monitoring; the reaction went to completion within 12 hours typically). The reaction mixture was concentrated *in vacuo* and dissolved in sodium citrate buffer (150 mM, 8 mL, adjusted to pH 7), containing $GdCl_3$ (260 mg, 0.99 mmol). The mixture was heated to 37 °C and carefully monitored by LC-MS. The chelation was complete after 5 hours. The reaction mixture was concentrated to 5 mL and purified by preparatory HPLC (method: 0-3 min: 90% H_2O (0.1% Formic Acid), 10% MeCN (0.1% Formic Acid). 2-23 min linear gradient to 95% MeCN. R_t= 13.9 min). The HPLC fractions were combined, the acetonitrile removed *in vacuo* followed by freeze-drying (white crystalline powder, 167 mg, 81%).

N.B. NMR characterisation could not be carried out due to the paramagnetic nature of gadolinium.

mp > 250 °C

ν_{max}(solid)/cm^{-1} 3271.7, 2930.7, 2160.5, 1600.0, 1506.5, 1364.9.

m/z (ESI) 1054.3145 ($C_{43}H_{55}GdN_6O_{15}$ requires 1054.3039)

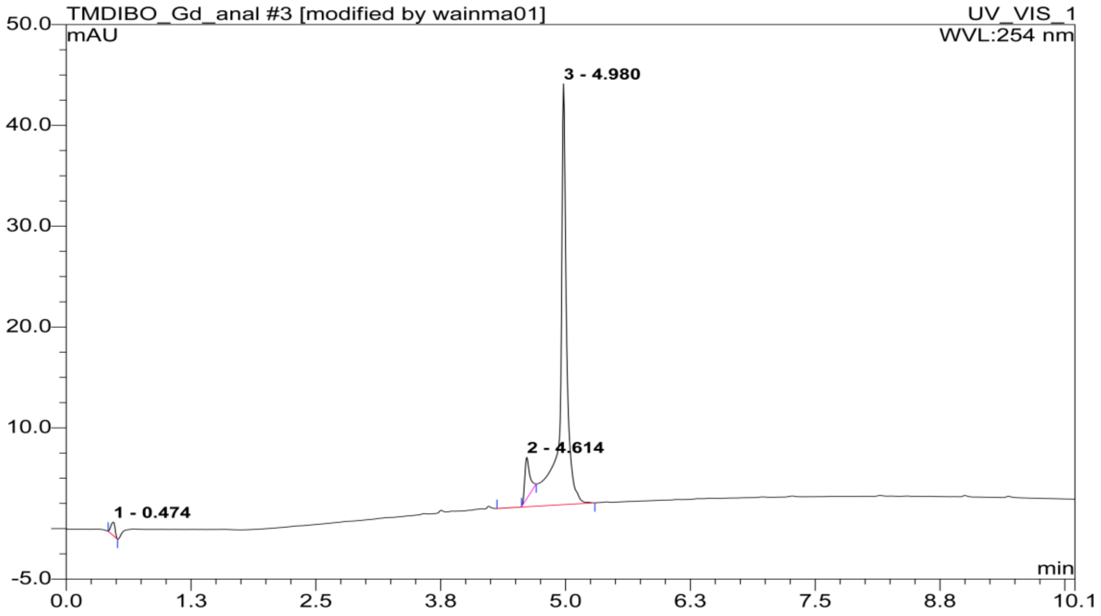

Analytical HPLC trace of TMDIBO-Lys-Gd, purified by semi-preparatory HPLC

To ensure that no free gadolinium cations were present after HPLC purification, xylenol orange was used as a metal indicator.[4]

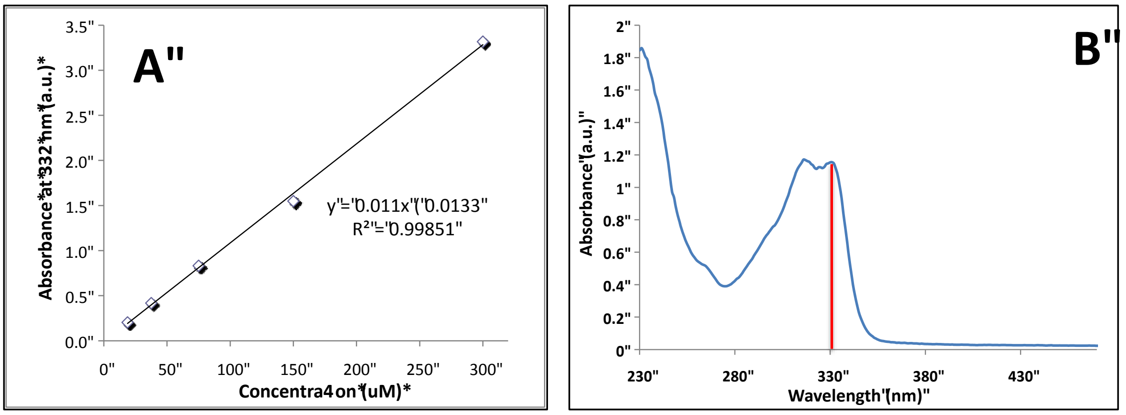

Fig. 1 Determining the concentration of TMDIBO-Lys-Gd **2** by UV. **A)** Absorbance values at 332 nm for a serial dilution of TMDIBO-Lys**. B)** Absorbance spectrum for TMDIBO-Lys-Gd **2**.

To determine the final probe concentration, a UV method was used based on the assumption that the UV absorbance of the probe would be the same as that of TMDIBO-Lys. Two absorbance peaks were observed for TMDIBO-Lys-Gd (Fig. 1B) and the peak

at 332 nm was chosen as it was more consistent in various samples. UV aborbances of a serial dilution of the TMDIBO-Lys were measured at this wavelength, and showed the expected linear relationship of absorbance with concentration (Fig. 1A). From this the concentration of the stock solution of TMDIBO-Lys-Gd was determined to be 79.8 mM, very close to the 79.3 mM concentration determined by mass (after HPLC purification). This probe solution in HBS buffer was used for further investigations on cells *in vitro* as well as imaging *in vivo.* The probe relaxivity was determined at 7 T in HBS buffer using a serial dilution of **2**.

III - *In vitro* methods

Cell Culture. Murine Lewis Lung adenocarcinoma (LL2) cells were purchased from ATCC and propagated (10^4–10^5/cm^2) in DMEM medium (Invitrogen), supplemented with 4.5 g/L of glucose, 2 mM L-glutamine and 10% fetal bovine serum (FBS, from PAA Laboratories). Cells were incubated with either **1** (50 µM, 24h, 37 °C) or solvent vehicle (PBS), centrifuged (400 g, 5 min, 4°C) to generate cell pellets (2 x 10^7 cells/pellet) and then reacted with **2** (1.0 mM, 45 mins, 37 °C). The cells were then washed in buffer (PBS containing 1% fetal bovine serum). T_1 relaxation rates R_1 (R_1=1/T_1) were measured at 7T.

MRI in vitro. Spin-lattice relaxation (T_1) maps were obtained using a 7.0T-horizontal bore MRI scanner (Agilent) with a 42 mm-volume coil for transmit and receive. An inversion-recovery spoiled gradient echo (IRSGE) sequence was used to generate T_1 maps from cell pellets using a hyperbolic-secant adiabatic inversion pulse and 18 inversion times between 0.1 and 25.6s (TR 4.52 ms; TE 2.16 ms; inter-scan delay 26s; field-of-view, 40mmx20mm; 128x32pts; 2 averages; 10° flip angle).

IV - *In vivo* methods

Mice. Five million LL2 cells were re-suspended in cold PBS and implanted in the lower flank (100 µl, s.c.) of BalbC nu/nu mice (Charles River). After seven days, animals were injected daily, for 3 days, with a solution of **1** (300 mg/kg, i.p.) in a mixture DMSO:PBS (1:9), or with solvent vehicle. On day 4, **2** was administered (250 µmol/kg, i.v.) and MR

imaging performed before, and at 2 and 24 h post injection. Twenty-four hours after administration of **2**, the mice were euthanized and a panel of tissues collected for *ex vivo* analysis of gadolinium concentration by ICP-MS. All animal experiments were conducted in accordance with the Animals (Scientific Procedures) Act of 1986 (UK) and were designed with reference to the UK Coordinating Committee on Cancer Research Guidelines for the Welfare of Animals in Experimental Neoplasia.[5] Protocols were approved by the Cancer Research UK, Cambridge Institute Animal Welfare and Ethical Review Body.

MRI *in vivo*. Animals were maintained under anesthesia at <2.5% isoflurane, using an Isoflo unit (Abbotts Laboratories Ltd), in 75% air/25% O_2 (2 L/min), heated with warm air to (37 °C) and monitored for breathing rate and body temperature (Biotrig, Small Animal Instruments). Coronal T_1 maps of mice were acquired with an IRSGE sequence (TR 5ms; TE 1.89ms; inter-scan delay 16s; 15 x 1.5 mm thick slices; field-of-view, 60x30mm; 128x64 data points, 2 averages, 10° flip angle with 8 different inversion times in the range 0.05-6.4s). A single 2 mm transversal slice was acquired through the tumor (TR, 4.16ms; TE, 1.91ms; inter-scan delay 12s; field-of-view, 30mmx30mm; 128x64 data points) with 2 averages and a 10° flip angle and the same inversion times as for the coronal images. T_2 weighted images were also acquired from coronal and transversal slices as positional references for the tumor and other tissues. T_1 map calculation was performed using a robust routine [6] implemented in Matlab (The Mathworks). 3D rendered maps of T_1 weighted images were generated using ImageJ.

V - *Ex vivo* methods

ICP-MS. Tissues (hind limb muscle, lung, skin, large intestine, pancreas, tumor, spleen, small intestine, heart, spleen, liver and kidney) were harvested from animals that had been injected with **1** or solvent vehicle and **2**, kept briefly on ice, weighed and then snap frozen in liquid N_2 for storage. Following thawing on ice, the tissues were homogenized in water (5 mL/g tissue) using a Precellys™ 24 system (Bertin Corp, Rockville, MD) using 3 cycles (600 rpm, 30 s, 4-10°C). Tissue homogenates were then hydrolyzed (16 h, conc. HCl, 1:1 vol), evaporated on a hot plate, the residue neutralized, re-suspended in citrate buffer (100 mM sodium citrate, pH 6.5), clarified by centrifugation (4 °C; 14,000g; 15 min) and stored at -20 °C. The hydrolysates were then freeze-dried and dissolved in 1 mL

ultrapure nitric acid (70%), prior to further digestion under microwawe heating at 180 °C for 20 min, using a Milestone MicroSYNTH Microwave labstation (Milestone Inc, Rockford, IL) equipped with an optical fiber temperature control and HPR-1000/6M six position high-pressure reactor. After digestion, the volume of each sample was brought to 3 mL with ultrapure water and the gadolinium content in each sample determined using inductively coupled plasma mass spectrometry (ICP-MS) (Element-2; Thermo-Finnigan, Waltham, MA). Three replicates of each sample solution were analyzed.

VI – Supplementary video 1

Imaging tissue glycosylation *in vivo* using MRI. 3D rendering of coronal T_1-weighted images.

VII - References

[1] A. A. Neves, H. Stöckmann, R. R. Harmston, H. J. Pryor, I. S. Alam, H. Ireland-Zecchini, D. Y. Lewis, S. K. Lyons, F. J. Leeper, K. M. Brindle, *FASEB J.* **2011**, *25*, 2528.

[2] A. A. Neves, H. Stockmann, Y. A. Wainman, J. C. Kuo, S. Fawcett, F. J. Leeper, K. M. Brindle, *Bioconjug Chem* **2013**, *24*, 934.; H. Stöckmann, A. A. Neves, S. Stairs, H. Ireland-Zecchini, K. M. Brindle, *Chem. Sci.* **2011**, *2*, 932.

[3] A. Barge, G. Cravotto, E. Gianolio, F. Fedeli, *Contrast Media Mol Imaging* **2006**, *1*, 184.

[4] P. Workman *et al.*, *Br J Cancer* **2010**, *102*, 1555.

[5] J. K. Barral, E. Gudmundson, N. Stikov, M. Etezadi-Amoli, P. Stoica, D. G. Nishimura, *Magn Reson Med* **2010**, *64*, 1057.
